# Supplementary material for: Characterization of a Novel African Swine Fever Virus p72 Genotype II from Nigeria
Source: Viruses. 2023 Apr 2;15(4):915. doi: 10.3390/v15040915 (PMC10146018; doi:10.3390/v15040915)
Supplement: Supplementary file 1 [file viruses-15-00915-s001.zip › viruses-2277667-supplementary.pdf]

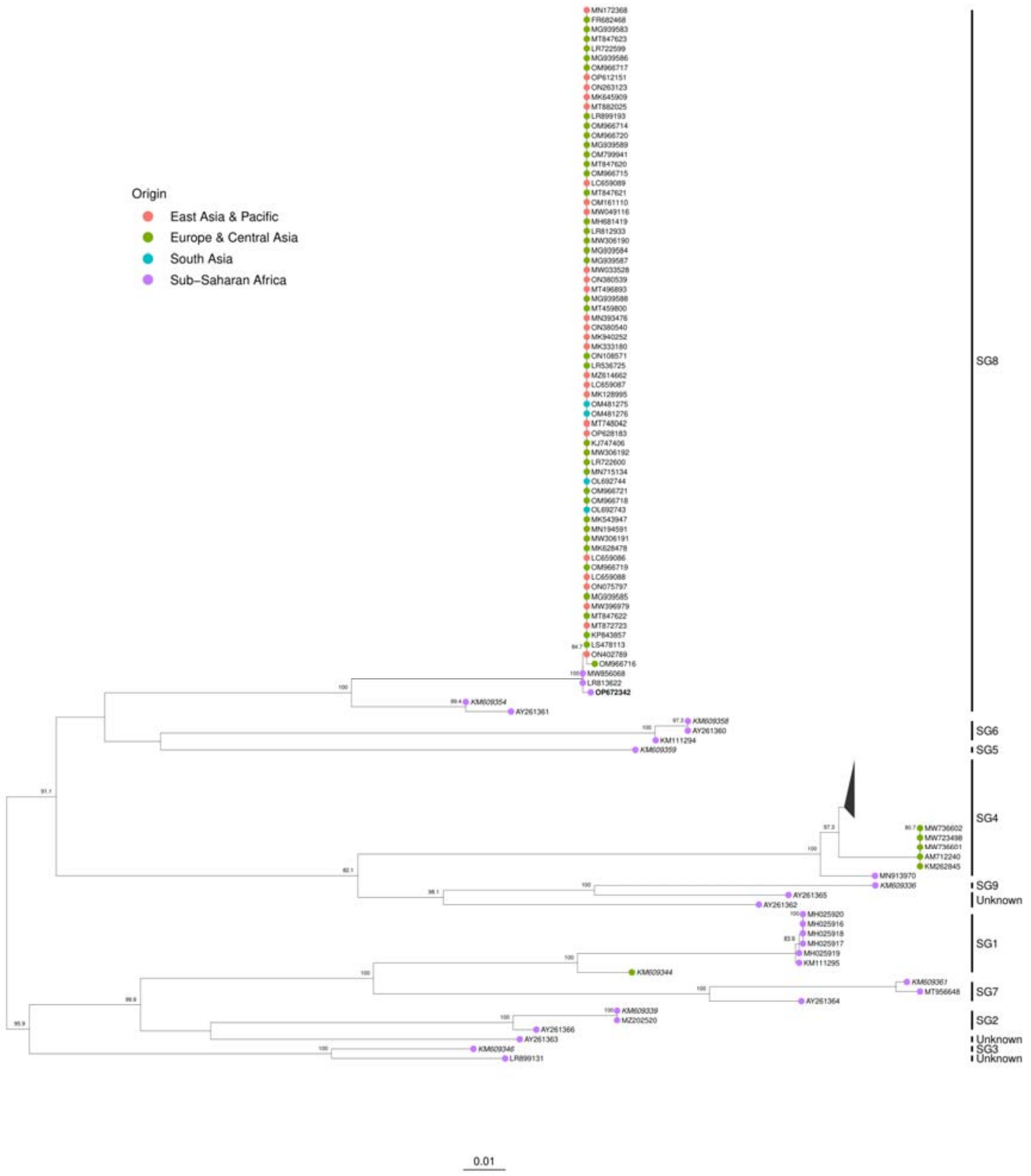

Figure S1: Phylogenetic comparison of ASFV strains on the full-length ASFV EP402R (CD2v) gene. Full length EP402R genes from 165 ASFV strains were analyzed by maximum-likelihood method with 100 bootstrap replicates. The phylogenetic tree was constructed using the IQ-TREE and visualized using ggtree. To reduce the size of the figure, one of the branches of SG4 was collapsed. Colored circles were used to indicate the geographical location of each strain. The scale bar indicates the number of substitutions per site.

Table S1. African swine fever whole genome sequences used for phylogenetic analysis, and their p72 genotypes and CDv2 serotypes.

Accession#	Isolate	Length (bp)	Host	Date	Origin	Genotype (B646L)	Serotype (EP402R)
MZ202520	K49	189523	Pig (domestic)	1949	Dem. Rep. of the Congo	I	2
KM262844	L60	182362	Pig (domestic)	1960	Portugal	I	4
KM262845	NHV	172051	Pig (domestic)	1968	Portugal	I	4
KP055815	BA71	180365	Pig (?)	1971	Spain: Badajoz	I	4
U18466	BA71V	170101	Vero adapted	1971	Spain	I	4
FN557520	E75	181187	Pig (domestic)	1975	Spain	I	4
MN270969	56/Ca/1978	183636	Pig (domestic)	1978	Italy: Sardinia	I	4
MW723480	Ca1978_2	181925	Pig (domestic)	1978	Italy: Sardinia	I	4
MN270970	57/Ca/1979	183639	Pig (domestic)	1979	Italy: Sardinia	I	4
MW723481	Nu1979	181859	Pig (domestic)	1979	Italy: Sardinia	I	4
ON185726	Dr-1980	183680	Pig (?)	1980	Dominican Republic	I	4
MN270971	139/Nu/1981	183645	Pig (domestic)	1981	Italy: Sardinia	I	4
MW800838	Or_1984	181924	Pig (domestic)	1984	Italy: Sardinia	I	4
MN270972	140/Or/1985	183723	Pig (domestic)	1985	Italy: Sardinia	I	4
MN270973	85/Ca/1985	181816	Pig (domestic)	1985	Italy: Sardinia	I	4
MW723482	Nu1986	181788	Pig (domestic)	1986	Italy: Sardinia	I	4
AM712240	OURT 88/3	171719	Tick (*O. erraticus*)	1988	Portugal	I	4
MN270974	141/Nu/1990	183720	Pig (domestic)	1990	Italy: Sardinia	I	4
MW723483	Nu1990_1	181751	Pig (domestic)	1990	Italy: Sardinia	I	4
MW723484	Nu1991_2	181741	Pig (domestic)	1991	Italy: Sardinia	I	4
MW723485	Nu1991_3	181725	Pig (domestic)	1991	Italy: Sardinia	I	4
MW723486	Nu1991_7	181750	Pig (domestic)	1991	Italy: Sardinia	I	4
MW723488	Nu1993_2	181748	Pig (domestic)	1993	Italy: Sardinia	I	4
MW723487	Or1993_1	181741	Pig (domestic)	1993	Italy: Sardinia	I	4
MN270975	142/Nu/1995	183724	Pig (domestic)	1995	Italy: Sardinia	I	4
MW723489	Nu1995_2	181753	Pig (domestic)	1995	Italy: Sardinia	I	4
MW723490	Nu1995_3	181740	Pig (domestic)	1995	Italy: Sardinia	I	4
MW723491	Nu1995_4	181697	Pig (domestic)	1995	Italy: Sardinia	I	4
MN270976	60/Nu/1997	181651	Pig (domestic)	1997	Italy: Sardinia	I	4
AM712239	Benin 97/1	182284	Pig (domestic)	1997	Benin	I	4
MN270977	26/Ss/2004	184581	Pig (domestic)	2004	Italy: Sardinia	I	4
MW723500	44076	181755	Pig (domestic)	2004	Italy: Sardinia	I	4
MW723496	74377	181753	Pig (domestic)	2004	Italy: Sardinia	I	4
MW723497	22649	181753	Pig (domestic)	2005	Italy: Sardinia	I	4
MW723495	72398 WB	181754	Pig (wild)	2005	Italy: Sardinia	I	4
MN270978	72407/Ss/2005	181699	Pig (domestic)	2005	Italy: Sardinia	I	4
MW723498	72912 WB	181804	Pig (wild)	2007	Italy: Sardinia	I	4
MW723499	22137	181751	Pig (domestic)	2008	Italy: Sardinia	I	4
MW788406	22943_2008	181733	Pig (domestic)	2008	Italy: Sardinia	I	4
MW723494	23221	181770	Pig (domestic)	2008	Italy: Sardinia	I	4

Accession#	Isolate	Length (bp)	Host	Date	Origin	Genotype (B646L)	Serotype (EP402R)
MW723493	46830	181746	Pig (domestic)	2008	Italy: Sardinia	I	4
KX354450	47/Ss/2008	184638	Pig (domestic)	2008	Italy: Sardinia	I	4
MW723492	4996 WB	181751	Pig (wild)	2008	Italy: Sardinia	I	4
KM102979	26544/OG10	182906	Pig (domestic)	2010	Italy: Sardinia	I	4
MW736612	31208	181684	Pig (domestic)	2011	Italy: Sardinia	I	4
MW736598	2019 WB	181742	Pig (wild)	2012	Italy: Sardinia	I	4
MW736603	63525 WB	181733	Pig (wild)	2012	Italy: Sardinia	I	4
MN270979	97/Ot/2012	184206	Pig (domestic)	2012	Italy: Sardinia	I	4
MW736608	113049 WB	181754	Pig (wild)	2013	Italy: Sardinia	I	4
MW736600	30322	181745	Pig (domestic)	2013	Italy: Sardinia	I	4
MW736607	32516	181758	Pig (domestic)	2013	Italy: Sardinia	I	4
MW736597	47039	181738	Pig (domestic)	2013	Italy: Sardinia	I	4
MW736601	49179 WB	181793	Pig (wild)	2013	Italy: Sardinia	I	4
MW736599	98039	181753	Pig (domestic)	2013	Italy: Sardinia	I	4
MN270980	22653/Ca/2014	181869	Pig (domestic)	2014	Italy: Sardinia	I	4
MW736605	51268	181753	Pig (domestic)	2014	Italy: Sardinia	I	4
MW736604	15998	181761	Pig (domestic)	2015	Italy: Sardinia	I	4
MW736610	28928	181738	Pig (domestic)	2015	Italy: Sardinia	I	4
MW736613	33747 WB	181753	Pig (wild)	2015	Italy: Sardinia	I	4
MW736609	6396 WB	181756	Pig (wild)	2015	Italy: Sardinia	I	4
MW736602	53706	181813	Pig (domestic)	2016	Italy: Sardinia	I	4
MW736606	34403	181759	Pig (domestic)	2017	Italy: Sardinia	I	4
MT932578	103917/18	181759	Pig (domestic)	2018	Italy: Sardinia	I	4
MT932579	55234/18	181761	Pig (domestic)	2018	Italy: Sardinia	I	4
MW736611	56140	181759	Pig (domestic)	2018	Italy: Sardinia	I	4
MW647171	LO2018 major	181758	Pig (domestic)	2018	Italy: Sardinia	I	4
MW647172	LO2018 minor	181758	Pig (domestic)	2018	Italy: Sardinia	I	4
LR812933	Arm/07/CBM/c2	190145	Pig (domestic)	2007	Armenia	II	8
FR682468	ASFV Georgia 2007/1	190584	Pig (domestic)	2007	Georgia	II	8
KJ747406	Kashino 04/13	189387	Pig (wild)	2013	Russia	II	8
MK628478	ASFV/LT14/1490	189399	Pig (wild)	2014	Lithuania	II	8
LS478113	Estonia 2014	182446	Pig (wild)	2014	Estonia	II	8
KP843857	Odintsovo_02/14	189333	Pig (wild)	2014	Russia	II	8
MH681419	ASFV/POL/2015/Podlaskie	189394	Pig (wild)	2015	Poland	II	8
MN194591	ASFV/Kyiv/2016/131	191911	Pig (domestic)	2016	Ukraine: Kyiv	II	8
MG939583	Pol16_20186_o7	189401	Pig (domestic)	2016	Poland	II	8
MG939584	Pol16_20538_o9	189399	Pig (domestic)	2016	Poland	II	8
MG939585	Pol16_20540_o10	189405	Pig (domestic)	2016	Poland	II	8
MG939586	Pol16_29413_o23	189393	Pig (domestic)	2016	Poland	II	8
LR722600	ASFV CzechRepublic 2017/1	190594	Pig (wild)	2017	Czech Republic	II	8
LR722599	ASFV Moldova 2017/1	190598	Pig (domestic)	2017	Moldova	II	8
MG939587	Pol17_03029_C201	189405	Pig (wild)	2017	Poland	II	8

Accession#	Isolate	Length (bp)	Host	Date	Origin	Genotype (B646L)	Serotype (EP402R)
MG939588	Pol17_04461_C210	189401	Pig (wild)	2017	Poland	II	8
MG939589	Pol17_05838_C220	189393	Pig (wild)	2017	Poland	II	8
MT847622	Pol17_31177_O81	189422	Pig (domestic)	2017	Poland	II	8
MT847620	Pol17_55892_C754	189414	Pig (wild)	2017	Poland	II	8
LR813622	Tanzania/Rukwa/2017/1	183186	Pig (domestic)	2017	Tanzania	II	8
OM799941	ASFV/Kaliningrad_17/WB-13869	189129	Pig (wild)	2017	Russia	II	8
MK645909	ASFV-wbBS01	189394	Pig (wild)	2018	China	II	8
LR536725	ASFV Belgium 2018/1	190599	Pig (wild)	2018	Belgium	II	8
MN715134	ASFV_HU_2018	190601	Pig (wild)	2018	Hungary	II	8
MK543947	Belgium/Etalle/wb/2018	190202	Pig (wild)	2018	Belgium	II	8
MK128995	China/2018/AnhuiXCGQ	189393	Pig (domestic)	2018	China	II	8
MT496893	GZ201801	189393	Pig (domestic)	2018	China	II	8
ON263123	GZ201801_2	189401	Pig (domestic)	2018	China	II	8
MK333180	Pig/HLJ/2018	189404	Pig (domestic)	2018	China	II	8
MT847621	Pol18_28298_O111	189409	Pig (domestic)	2018	Poland	II	8
OM966720	ASFV/Kaliningrad_18/WB-12516	189143	Pig (wild)	2018	Russia	II	8
OM966714	ASFV/Kaliningrad_18/WB-12523	189111	Pig (wild)	2018	Russia	II	8
OM966715	ASFV/Kaliningrad_18/WB-12524	189133	Pig (wild)	2018	Russia	II	8
OM966721	ASFV/Kaliningrad_18/WB-9734	189127	Pig (wild)	2018	Russia	II	8
OM966716	ASFV/Kaliningrad_18/WB-9735	189129	Pig (wild)	2018	Russia	II	8
OM966717	ASFV/Kaliningrad_18/WB-9763	189125	Pig (wild)	2018	Russia	II	8
OM966718	ASFV/Kaliningrad_18/WB-9766	189129	Pig (wild)	2018	Russia	II	8
OM105586	LYG18	188389	Pig (?)	2018	China	II	
MW033528	ASFV-wbShX01	189401	Pig (wild)	2019	China	II	8
MW049116	ASFV Korea/pig/Yeoncheon1/2019	190598	Pig (domestic)	2019	South Korea	II	8
MN393476	ASFV Wuhan 2019-1	190576	Pig (domestic)	2019	China	II	8
MW306190	ASFV/Amur 19/WB-6905	189248	Pig (wild)	2019	Russia	II	8
MT459800	ASFV/Kabardino-Balkaria 19/WB-964	189252	Pig (wild)	2019	Russia	II	8
MT748042	ASFV/Korea/pig/PaJu1/2019	190597	Pig (domestic)	2019	South Korea: PaJu	II	8

Accession#	Isolate	Length (bp)	Host	Date	Origin	Genotype (B646L)	Serotype (EP402R)
MN172368	ASFV/pig/China/CAS19-01/2019	189405	Pig (?)	2019	China: Zhuhai	II	8
MW306191	ASFV/Primorsky 19/WB-6723	189256	Pig (wild)	2019	Russia	II	8
MW396979	ASFV/Timor-Leste/2019/1	192237	Pig (domestic)	2019	Timor-Leste	II	8
MW306192	ASFV/Ulyanovsk 19/WB-5699	189263	Pig (wild)	2019	Russia	II	8
MK940252	CN/2019/InnerMongolia-AES01	189403	Pig (wild)	2019	China	II	8
MW856068	MAL/19/Karonga	183325	Pig (domestic)	2019	Malawi	II	8
MT847623	Pol19_53050_C1959/19	189413	Pig (wild)	2019	Poland	II	8
OM966719	ASFV/Kaliningrad_19/WB-10168	189131	Pig (wild)	2019	Russia	II	8
MZ614662	CADC_HN09	190257	Pig (?)	2019	China	II	8
ON075797	Korea/YC1/2019	188950	Pig (wild)	2019	South Korea	II	8
MT872723	VN/HY-ASFV1(2019)	189113	Pig (domestic)	2019	Viet Nam	II	8
MT882025	VN/QP-ASFV1(2019)	189081	Pig (?)	2019	Viet Nam	II	8
LC659086	AQS-C-1-21	189398	Pig (?)	2019/2020	China	II	8
LC659087	AQS-C-1-22	189405	Pig (?)	2019/2020	China	II	8
LC659089	AQS-P-201202	189401	Pig (?)	2019/2020	The Philippines	II	8
LC659088	AQS-P-20901-1	189396	Pig (?)	2019/2020	The Philippines	II	8
LR899193	ASFV Germany 2020/1	190592	Pig (wild)	2020	Germany	II	8
MW521382	HuB20	188643	Pig (domestic)	2020	China	II	
OL692744	IND/AR/SD-61/2020	190572	Pig (domestic)	2020	India	II	8
OL692743	IND/AS/SD-02/2020	190517	Pig (domestic)	2020	India	II	8
MW656282	Pig/Heilongjiang/HRB1/2020	189355	Pig (domestic)	2020	China	II	
OP672342	RV502	185318	Pig (domestic)	2020	Nigeria	II	8
OM481275	ABTCVSCK_ASF001	190598	Unknown	2020	India: Meghalaya	II	8
ON380539	HB03A	189737	Pig (domestic)	2020	China	II	8
ON380540	HB31A	189456	Pig (domestic)	2020	China	II	8
OP628183	Korea/HC224/2020	188645	Pig (wild)	2020	South Korea	II	8
OM161110	SY-1	189404	Pig (wild)	2020	China	II	8
ON402789	ASF/VN/CanTho-OM/2021	189487	Pig (domestic)	2021	Viet Nam	II	8
ON400500	YNFN202103	178014	Pig (domestic)	2021	China	II	
OM481276	ABTCVSCK_ASF007	190595	Unknown	2021	India: Assam	II	8
OM105587	JX21	181147	Pig (?)	2021	China: Jiangxi	II	
OP612151	SY-2	189404	Pig (domestic)	2021	China: Wuhan	II	8

**Accession#**	**Isolate**	**Length (bp)**	**Host**	**Date**	**Origin**	**Genotype (B646L)**	**Serotype (EP402R)**
ON108571	2802/AL/2022 Italy	190596	Pig (wild)	2022	Italy: Piedmont	II	8
AY261365	Warmbaths	190773	Tick (?)	1987	South Africa	III	Unknown
AY261366	Warthog	186528	Warthog	1980	Namibia	IV	2
KM111295	Ken06.Bus	184368	Pig (domestic)	2006	Kenya	IX	1
MH025919	N10	188611	Pig (domestic)	2015	Uganda	IX	1
MH025918	R25	188630	Pig (domestic)	2015	Uganda	IX	1
MH025920	R35	188629	Pig (domestic)	2015	Uganda	IX	1
MH025917	R7	188628	Pig (domestic)	2015	Uganda	IX	1
MH025916	R8	188627	Pig (domestic)	2015	Uganda	IX	1
AY261364	Tengani 62	185689	Pig (domestic)	1962	Malawi	V	7
AY261362	Mkuzi 1979	192714	Tick (*O. sp.*)	1979	South Africa	VII	Unknown
MN913970	Liv13/33 (OmLF2)	188277	Tick (*O. moubata*)	1983	Zambia	VII	Unknown
AY261361	Malawi Lil-20/1 (1983)	187612	Tick (*O. moubata*)	1983	Malawi	VIII	8
AY261360	Kenya 1950	193886	Pig (domestic)	1950	Kenya	X	6
KM111294	Ken05/Tk1	191058	Tick (*O. moubata*)	2005	Kenya	X	6
MW856067	BUR/18/Rutana	176564	Pig (domestic)	2018	Burundi	X	
LR899131	ASFV Ken.rie1	189950	Tick (*O. moubata*)	2019	Kenya	X	Unknown
MT956648	Uvira B53	180916	Pig (domestic)	2019	Dem. Rep. of the Congo	X	7
AY261363	Pretoriuskop/96/4	190324	Tick (*O. moubata*)	1996	South Africa	XX	Unknown
